# Supplementary material for: Processing of a single ribonucleotide embedded into DNA by human nucleotide excision repair and DNA polymerase η
Source: Sci Rep. 2019 Sep 26;9:13910. doi: 10.1038/s41598-019-50421-8 (PMC6763444; doi:10.1038/s41598-019-50421-8)
Supplement: Supplementary file 1 — Supplementary Figure S1 [file 41598_2019_50421_MOESM1_ESM.docx]

**Supplementary Information**

**Processing of a single ribonucleotide embedded into DNA by human nucleotide excision repair and DNA polymerase η**

Akira Sassa^a*^, Haruto Tada^b^, Ayuna Takeishi^a^, Kaho Harada^a^, Megumi Suzuki^a^, Masataka Tsuda^c^, Hiroyuki Sasanuma^d^, Shunichi Takeda^d^, Kaoru Sugasawa^b^, Manabu Yasui^e^, Masamitsu Honma^e^ and Kiyoe Ura^a^

^a^Department of Biology, Graduate School of Science, Chiba University, Chiba 263-8522, Japan

^b^Biosignal Research Center, Kobe University, 1-1 Rokkodai-cho, Nada-ku, Kobe 657-8501, Japan

^c^Program of Mathematical and Life Science, Graduate School of Integrated Science for Life, Hiroshima University, Higashi-Hiroshima 739-8526, Japan

^d^Department of Radiation Genetics, Graduate School of Medicine, Kyoto University, Yoshida Konoe, Sakyo-ku, Kyoto 606-8501, Japan

^e^Division of Genetics and Mutagenesis, National Institute of Health Sciences, 3-25-26 Tonomachi, Kawasaki-ku, Kawasaki-shi, Kanagawa 210-9501, Japan

***Corresponding author:** Akira Sassa, Department of Biology, Graduate School of Science, Chiba University, Chiba 263-8522, Japan. TEL: +81-43-290-2776, FAX: +81-43-290-2776; E-mail address: a-sassa@chiba-u.jp

**Figure S1** Reconstituted human nucleotide excision repair excises oxidized ribonucleotide. The substrates containing UV-induced 6-4-photoproducts (6-4PP), dG, 8-oxo-dG, rG, and 8-oxo-rG were internally labeled with ^32^P and used for the dual incision assay. The DNA samples were subjected to 10% denaturing polyacrylamide gel electrophoresis followed by autoradiography. [M] ^32^P-labeled 25-nucleotides (nt) ladder.
